# Why Don’t the Mutant Cells That Evade DNA Repair Cause Cancer More Frequently? Importance of the Innate Immune System in the Tumor Microenvironment

**DOI:** 10.3390/ijms24055026

**Published:** 2023-03-06

**Authors:** Shubhasmita Mohapatra, Jared Cafiero, Khosrow Kashfi, Parag Mehta, Probal Banerjee

**Affiliations:** 1 Department of Chemistry, The College of Staten Island, City University of New York, Staten Island, NY 10314, USA; 2 Department of Molecular, Cellular and Biomedical Sciences, Sophie Davis School of Biomedical Education, City University of New York School of Medicine, New York, NY 10031, USA; 3 Graduate Program in Biology, City University of New York Graduate Center, New York, NY 10016, USA; 4 Aveta Biomics, Inc., 110 Great Road, Suite 302, Bedford, MA 01730, USA

**Keywords:** glioblastoma, tumor microenvironment, macrophages, repolarization, chemokines, immunotherapy

## Abstract

The standard of care for most malignant solid tumors still involves tumor resection followed by chemo- and radiation therapy, hoping to eliminate the residual tumor cells. This strategy has been successful in extending the life of many cancer patients. Still, for primary glioblastoma (GBM), it has not controlled recurrence or increased the life expectancies of patients. Amid such disappointment, attempts to design therapies using the cells in the tumor microenvironment (TME) have gained ground. Such “immunotherapies” have so far overwhelmingly used genetic modifications of Tc cells (Car-T cell therapy) or blocking of proteins (PD-1 or PD-L1) that inhibit Tc-cell-mediated cancer cell elimination. Despite such advances, GBM has remained a “Kiss of Death” for most patients. Although the use of innate immune cells, such as the microglia, macrophages, and natural killer (NK) cells, has been considered in designing therapies for cancers, such attempts have not reached the clinic yet. We have reported a series of preclinical studies highlighting strategies to “re-educate” GBM-associated microglia and macrophages (TAMs) so that they assume a tumoricidal status. Such cells then secrete chemokines to recruit activated, GBM-eliminating NK cells and cause the rescue of 50–60% GBM mice in a syngeneic model of GBM. This review discusses a more fundamental question that most biochemists harbor: “since we are generating mutant cells in our body all the time, why don’t we get cancer more often?” The review visits publications addressing this question and discusses some published strategies for re-educating the TAMs to take on the “sentry” role they initially maintained in the absence of cancer.

## 1. Introduction

Glioblastoma (GBM) is one of the deadliest forms of cancer, with an average life expectancy of about 14–18 months from detection [1]. The standard of care (SOC) for GBM is surgical resection of the tumor followed by chemo and or radiation therapy to eliminate the residual cancer cells [2,3]. However, in most cases, GBM returns soon after the SOC and eventually overcomes the patient. In chemotherapy, the general strategy has been the use of antimetabolites that inhibit DNA replication and other compounds that target specific signaling proteins that are often overactivated by mutations [4]. Unfortunately, such chemotherapeutic agents also inhibit the normal signaling proteins needed by healthy cells, such as the immune cells, thus causing severe side effects linked to lymphopenia. On the other hand, though targeted to cancer cells, radiation therapy kills the juxtaposed normal cells, such as the microglia, macrophages, and the infiltrating immune cells, thereby weakening the overall system. Overall, such therapeutic strategies have not significantly extended the life of GBM patients.

Despite the ongoing effort to develop effective therapeutic strategies, an important question remains: “biochemical knowledge reveals that, despite effective DNA repair, we are constantly generating some cells with mutant DNA molecules, so why don’t we, relatively speaking, develop cancer more frequently?” A general belief is that our vigilant immune cells promptly eliminate such mutant cells. However, when our immune system is weakened, the mutant cells can proliferate to precipitate cancer, especially in the later years. We found hard evidence corroborating this belief in a study conducted by Afshar-Sterle and coworkers, which showed that the loss of the tumor-suppressor function of the gene *BLIMP1* or deregulated expression of the *BCL6* oncogene occurs in a large portion of B-cell lymphomas in human patients, however, the deliberate introduction of Blimp1 deficiency or Bcl6 overexpression in the B cells of mice does not precipitate lymphoma unless the T-cell receptor CD28- and Fas-ligand activities are simultaneously impaired in the CD8+ Tc cells [5]. Additionally, consistent with the hypothesis that cells with mutated DNA are eliminated by microglia and macrophages, Shi and coworkers observed that consequent to *Knl1* deletion, neural progenitor cells accumulate DNA damage on mis-segregated chromosomes in the mitotic spindle, which triggers apoptosis and phagocytosis by the microglia [6]. However, Kasapi and Triantafyllopoulou note that the “role of genotoxic stress as an instructor of macrophage-mediated immune defense and tissue remodeling is only beginning to be understood” [7]. Therefore, more research is required to elevate the currently-held belief to a widely-accepted phenomenon.

## 2. The Dichotomous Role of Tumor-Associated Microglia and Macrophages and Possible Triggers to Turn Them against the GBM Tumor

During the last decade, a significant focus has been placed on the adaptive immune system as a tool to eliminate cancer cells. This has resulted in the development of cytotoxic T-cell (Tc)-based immunotherapy [8], which has shown considerable success in several cases of melanoma [9,10], but, unfortunately, not for GBM and some peripheral cancers, such as endometrial/ovarian, pancreatic, liver, and colon cancers, to name a few. Furthermore, adverse events, mainly due to autoimmune reactions, have been reported following Tc-based immunotherapy [11]. As for the immediately-acting innate immune cells such as microglia and macrophages, they are recruited into the GBM tumor and changed from the tumoricidal, “classically-activated” “M1”-type to a tumor-promoting, “alternatively-activated” “M2” phenotype by cytokines secreted by the GBM cells [12]. Thus, the GBM microenvironment harbors mostly M2-type tumor-associated microglia/macrophages (TAMs), very few M1-type microglia, and some nonactivated M0-type microglia [12]. Since the direct killing of GBM cells has proven to be ineffective in eliminating all tumor cells and reliably preventing cancer relapse, an attractive strategy could be to re-educate the M2-type microglia or macrophages in the tumor microenvironment (TME) to the M1-type, thereby launching a Trojan horse-like attack from inside the tumor. A few such strategies have been discussed here.

## 3. Tools to Topple the STAT-3/STAT-1 Balance in the Microglia and Macrophages and Kill the GBM and GBM Stem Cells

The M2 state of the microglia and macrophages is centrally controlled by the transcription factor STAT-3, which is known to stimulate the expression of immune-suppressive cytokines like IL-10, IL-4, and IL-13 [13]. In addition, it upregulates the expression of the key enzyme Arginase-1 (Arg-1) that marks the M2-type microglia and macrophages [12,13,14]. The cytokine IL-10 causes inhibition of STAT-1 by suppressing the phosphorylation of this transcription factor [12,14]. Therefore, the inhibition of STAT-3 would cause an activation of STAT-1 and subsequent STAT-1-mediated events such as the induction of inducible nitric oxide synthetase (iNOS; also known as NOS2), MCP-1, and IL-12, which is typical of M1-type microglia and macrophages [12,14,15,16]. Furthermore, we know from earlier studies that upon release from the microglial cells in the brain, the chemokine MCP-1 (a.k.a. CCL2) crosses the blood-brain barrier into the peripheral system to bind to its receptor, CCR2, expressed by activated macrophages and natural killer (NK) cells, and thereby cause recruitment of these cells into the GBM tumor in the brain [15,16,17]. Thus, the inhibition of STAT-3 in the microglia is central to a process that links to the recruitment of an army of M1 macrophages and activated NK cells into the GBM to eliminate GBM cells and GBM stem cells [15,16,18,19,20].

Due to the mechanisms discussed in the previous section, finding or designing agents to inhibit STAT-3 has been a popular strategy among researchers keen on developing therapies against cancer [21]. Yet no FDA-approved STAT-3 inhibitor is available currently. Several natural products (mostly polyphenols and antioxidants) that inhibit STAT-3 have been used in preclinical studies against various types of cancer [22]. We have shown in a series of publications that curcumin (CC) and synergistic formulations of CC and other polyphenols, such as resveratrol (Res) and epicatechin gallate (ECG) (TriCurin), can inhibit STAT-3 in the microglia and macrophages in GBM as well as HPV+ cervical cancer, thereby repolarizing these cells in the TME to the M1 phenotype [15,19,20,23]. Almost all chemotherapeutic agents (CAs) are designed to block DNA replication in fast-dividing cells such as cancer cells. Intriguingly, at least one, paclitaxel (Taxol), functions by blocking the microtubule-dependent cell division machinery. Additionally, this same chemotherapeutic agent (Taxol) is known to inhibit cytokine-mediated STAT-3 activation and its interactions with microtubules [24]. Therefore, the efficacy of Taxol in eliminating tumor cells through the repolarizing of the TAMs from the M2 to M1 type deserves further investigation.

## 4. Debate over Arginase-1 Expression by Tumor-Associated Microglia/Macrophages in Humans versus Mice

As mentioned in the previous section, Arg-1 is highly expressed by M2-type microglia and macrophages. This urea cycle enzyme is believed to deplete the amino acid arginine, which is also a substrate for the enzyme iNOS that is highly expressed by the M1-type microglia and macrophages [12,15,19,23]. iNOS uses arginine as its substrate to generate nitric oxide (NO), a crucial signaling molecule that combines with reactive oxygen species to generate cytotoxic, reactive nitrogen species inside the tumor [25], thereby eliminating cancer cells and cancer stem cells. A high Arg-1 expression in the M2 microglia and macrophages is expected to disrupt the supply of arginine to iNOS, thus inhibiting the generation of NO and reactive nitrogen species. Therefore, Arg-1 expression by TAMs is a critical event determining their polarization states. Yet, currently, there is considerable debate over using Arg-1 to mark the activation state of human microglia and macrophages [26,27]. It seems that although mouse monocytes show IL-4-evoked induction of Arg-1, this is not observed in human monocytes [26,28]. The literature on the induction of Arg-1 by interleukins is replete with many mechanisms, and Makita and coworkers report that human IL-10 augments IL-4-mediated induction of Arg-1 in monocytes [29]. Furthermore, Kupani and coworkers observed IL-10- and TGFβ-induced expression of Arg-1 in human monocytes [30]. To address this dichotomy, Thomas and Mattila state that cultured monocytes from various sources can elicit responses that are different from macrophage responses in vivo [27]. Their second but legitimate argument is that these debating groups had attempted to identify the Arg-1 protein instead of measuring its activity. However, most biochemists will agree that the apparent absence of a protein is not a full-proof sign of non-expression of the enzyme mainly due to the differing sensitivities of the antibodies and the high V_max_ value of Arg-1, which, therefore, can produce ornithine at very low concentrations. Cognizant of this controversy, all of our studies of M2 → M1 repolarization of TAMs have used in vivo analysis using either immunohistochemistry (IHC) or flow cytometry analysis of dissociated tumor cells after fixing and antibody staining [15,16,19,20,23]. Thus, it is likely that data from mice and humans would be similar if the experiments were conducted on intact tumor tissue rather than cultured monocytes.

## 5. Recruitment of Activated Natural Killer (NK) Cells into the GBM Tumor

Among the innate immune cells, interferon gamma (IFNγ)-activated NK cells are known to play a crucial role in eliminating cancer cells and cancer stem cells [16,20]. The mechanisms of NK cell-mediated elimination of microglia and macrophages have been studied earlier. Thus, Lunemann and coworkers used human microglia and human NK cells to show that IL-2-activated NK cells formed immune-synapses with resting (M0) microglia to kill them but sparing the lipopolysaccharide (LPS)-activated microglia (M1) [31]. This microglia recognition occurred mainly through the NK-cell-harbored receptor proteins NKG2D and NKp46 since the antibodies to these proteins blocked the killing completely. Furthermore, MHC class I molecules modestly expressed by the microglia appeared to abrogate NK-cell-mediated killing due to toll-like receptor 4 (TLR4) stimulation by LPS, thus protecting these microglial cells. Intriguingly, in vitro cultured peripheral blood monocyte-derived macrophages were not protected from the NK cells following LPS activation. Based on the ability of NK cells to eliminate tumor cells, they have been considered for use in clinical trials involving immunotherapy [32,33,34,35,36].

Although it is accepted that NK cells are recruited into the GBM tumor, how they are drawn into the brain has been an important question, with multiple chemokines proposed to be involved by various research teams. A study showing NK cell chemotaxis into the liver during infection noted the involvement of the chemokine MIP-1a (a.k.a. CCL3) [37]. Morrison and coworkers observed that CCL2 was involved in NK-cell recruitment into the lungs during aspergillosis [38], and Hokerness and coworkers showed that this NK-cell recruitment required CCL2 plus its receptor, CCR2 [39]. Additionally, Trifilo and coworkers report that CXCL10 promotes innate defense against coronavirus infection by recruiting and stimulating NK cells [40]. In our studies in the GBM mouse model, we have observed that repolarization of the TAMs from M2 to the M1 state is associated with a dramatic increase in the expression of CCL2 (a.k.a MCP-1) in the microglia/macrophages, which is concomitant with the recruitment of activated NK cells into the TME [15]. Earlier research has demonstrated that CCL2 is expressed as a marker by M1 microglia and macrophages [41,42]. Furthermore, CCL2 reportedly can compromise the blood–brain barrier (BBB) and translocate from the brain to the peripheral system, thereby affecting recruitment of immune cells such as M1-type macrophages and NK cells, which express the CCL2 receptor CCR2 [43,44,45,46]. Based on these findings, we have proposed that after the initial repolarization of the microglia in the GBM TME from M2 → M1 in a syngeneic mouse model after curcumin treatment, CCL2 released by the TAMs causes intratumor recruitment of activated M1-type macrophages and IL-12-activated NK cells from the periphery [15].

**Figure 1:**
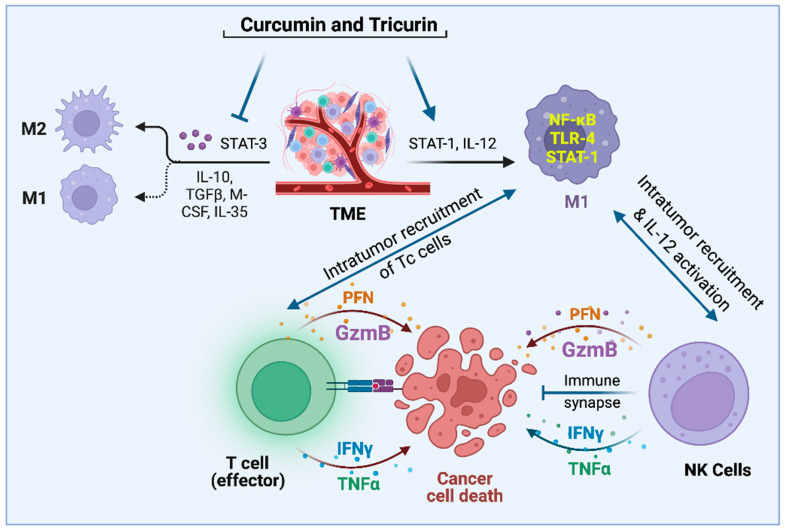
Pleiotropic natural agents topple the STAT-3/STAT-1 balance in the TAMs and recruit NK and Tc cells into the TME, thus launching an attack on tumor cells and tumor stem cells [15,19,20,23,31].

Once inside a tumor, the role of activated NK cells in eliminating tumor cells has been more generally accepted. As mentioned earlier, NK cell-based immunotherapy has been considered for clinical trials [32,33,34,35,36]. In the clinical application of NK cell therapy, deliberate intratumor infusion of NK cells is followed by IL-2 administration to activate the introduced NK cells. However, several factors render in vivo IL-2-mediated activation of NK cells a risky strategy. In addition to toxicity due to IL-2 administration, this causes the proliferation of immunosuppressive regulator T (T_reg_) cells [47]. Therefore, NK cell therapy has relied on in vitro IL-2 activation of NK cells followed by infusion of the activated cells. In our syngeneic mouse models of GBM and human papillomavirus (HPV)-mediated cancer, we have consistently observed the recruitment of activated NKp46+ NK cells and Tc cells into a tumor in mice treated with curcumin or a synergistic formulation containing curcumin, resveratrol, and epicatechin gallate, Tricurin (Figure 1) [15,19,20]. During our studies in the GBM mice, we also discovered an additional property of NK cells. NK cell recruitment was responsible partly for the curcumin-triggered repolarization of the TAMs from M2- to M1-type [15]. The intriguing offshoot of our studies is that both curcumin and Tricurin appear to be safe tools that can replace IL-2 in causing the activation and intratumor recruitment of NK cells [15,19]. Taken together, safe strategies appear to be available to turn both the TAMs and the NK cells against tumor cells that may have acquired diverse mutations in the process of becoming malignant.

## 6. Multiple Strategies of Immunotherapy and the Involvement of the Innate Immune System

Currently, immunotherapy, popularly known as “immune checkpoint inhibitor therapy,” mainly refers to a strategy of empowering CD8+, cytotoxic Tc cells of the adaptive immune system to eliminate cancer cells [48]. In order to prevent autoimmune attacks, the antigen-presenting cells of an organism express program cell death ligands (PD-L1), PD-L2, as well as the major histocompatibility complex (MHC), which bind to the protein program cell death one (PD-1) and the TCR/CD3 complex, respectively, thereby dampening the cytotoxicity of the Tc cells [48]. Most cancer cells also express high levels of PD-L1 to evade attack by Tc cells. Currently, two FDA-approved PD-1 antibody-based immunotherapy drugs are marketed under the names, Keytruda and Opdivo [49,50]. Additionally, the Tc cells express a protein, receptor protein cytotoxic T lymphocyte antigen four (CTLA4), which binds to ligands CD80 and CD86 expressed by antigen-presenting cells and cancer cells [8]. This CTLA4–CD80/CD86 interaction antagonizes the interaction of the Tc antigen CD28 with CD80/CD86, which activates the Tc cells. Therefore, CTLA4 inhibition would cause the activation of Tc cells against cancer cells. To achieve this, the FDA-approved, antibody-based drug Yervoy has been used for various types of cancer, including melanoma [51]. Another potential candidate protein to be included in immunotherapy is COP9 signalosome 5 (CSN5). Lim and coworkers have shown that CSN5, which is induced by nuclear factor kappa B (NF-κB) p50:p65 heterodimer (NF-κB p65), is required for tumor necrosis factor alpha (TNFα)-mediated stabilization of PD-L1 in cancer cells [52]. In their study, curcumin-evoked inhibition of CSN5 caused a decrease in PD-L1 expression in cancer cells, sensitizing them to anti-CTLA4 therapy. Intriguingly, curcumin also inhibits CTLA-4 [53]. Therefore, the inclusion of CSN5 as a target could increase the efficacy of immunotherapy. Finally, a newly invented strategy involving “Base Editing” appears to have given some leukemia patients a new lease on life [54]. Developed six years ago by David Liu, this technique of base editing uses a mutated version of the CRISPR Cas9 protein to target DNA sequences containing a “C” or an “A” to convert them through deamination to U and inosine (I), respectively. Coupling this step with inhibitors of the base excision repair enzymes enabled Liu and coworkers to produce mutations that can correct or introduce pathogenic changes [55]. Using this strategy and allogenic Tc cells, mutant Tc cells were created that could eliminate malignant and normal Tc cells of a leukemia patient, thus making the patient cancer-free [54]. Typically, this step is followed by a transfer of healthy bone marrow-derived T cells to the patient.

As for immunotherapy for glioblastoma in particular, most attempts have yielded only limited success [56]. Nevertheless, experimental evidence has suggested that manipulating the innate immune system might be beneficial. The discussion above shows that immunotherapy has so far involved the adaptive immune system. Two questions remain: (one) does the innate immune system influence the adaptive immune system, and does manipulating the adaptive immune system enhance the immunotherapy in use so far? and (two) has the innate immune system been considered as a primary mode of attack on cancer cells? Answers to the first question come from studies on dendritic cells (DCs), which, as innate immune cells, are known to be involved in the recruitment and activation of the Tc cells. Unlike the macrophages, which are recruited into the brain, likely by chemokines, the DCs, though not present in the brain parenchyma, are concentrated in the blood vessel-rich regions around the ventricles, such as choroid plexus and meninges [57,58,59]. From these niches, the DCs migrate to the brain and spinal cord under pathological conditions via lymphatic ducts or blood capillaries [60]. Among the different types of DC cells, the plasmacytoid DCs (pDCs) recognize pathogens such as viruses through toll-like receptor TLR7- and TLR9-signaling and secrete type 1 interferons (IFN1), which strongly activate CD8+ Tc cells [61,62,63]. Intriguingly, similar to the M1-type microglia and macrophages, the DCs respond to inflammation and infection by secreting inflammatory cytokines like IL-6 and IL-12 and chemokines CCL3, CCL4, CXCL8, and CXCL10 to recruit immune cells [64]. Similar to the DCs, the microglia are also known to cause recruitment of T cells from the periphery, although through a mechanism that involves the noncanonical nuclear factor κB (NF-κB)-inducing kinase (NIK) [65]. Additionally, other researchers have reported reciprocal signaling between the CNS microglia and the effector Tc cells in the context of neurodegenerative diseases and glioblastoma [66,67], and general surveillance of the CNS [68]. Thus, antigen-presenting cells such as DCs and microglia can regulate the recruitment and activation of Tc cells. The innate immune cells, like engineered DCs and activated NK cells, have been used in glioblastoma therapy [33,34,36,69]. It appears from the observations of repolarization of the microglia and the macrophages, intratumor recruitment of NK cells, and inhibition of CSN5 [15,16,19,20,23,52], that involvement of the innate immune system to assist the adaptive immune system may yield an effective and safe strategy of eliminating GBM as well as other solid tumors. Finally, question (two) has been answered in therapeutic applications of dendritic cells and NK cells, as discussed before [33,34,36,69].

Due to our particular interest in GBM therapy using microglia and macrophages, we next focused our attention on the use of these antigen-presenting innate immune cells. Reports of using macrophages in cancer therapy appear to be undergoing explosive growth. These studies can be classified roughly into two groups: (one) elimination of tumor-promoting TAMs (M2-type) and the inhibition of further recruitment into the tumor, and (two) reprogramming of the M2 TAMs into the tumoricidal M1 TAMs. However, the more effective antitumor treatments appear to involve a combination of traditional chemotherapy with the targeting of TAM, followed by the emerging immunotherapy involving immune checkpoint inhibition by targeting PD-1, PD-L1, or CTLA4, as discussed earlier.

Inhibition of macrophage recruitment into tumors to inhibit M2-type macrophage-evoked tumor progression and metastasis was attempted by blocking some chemokine signaling pathways that cause the intratumor recruitment of macrophages. Such chemokine signaling involved the receptors for CCL2 (CCR2), CCL5 (CCR5), and CXCL12 (CXCR4) [70], which also enhanced STAT-3 activity and M2 polarization of macrophages and retention inside the tumor [71,72]. Fourteen clinical trials targeting the CCL2/CCR2 axis using five investigational drugs have been conducted with disappointing results. One of these drugs (BMS813160) is currently in Phase II trials for colorectal and pancreatic cancer [73,74,75]. The disappointing outcome was attributed to improper patient selection. It was felt that patients selected for high CCR2 expression in their tumors would have shown a more robust response in the clinical trials. Selective inhibition of a single, specific target with drugs has been attempted in most clinical studies, however, they have yielded inconsistent results and precipitated many side effects. For example, the CCL2 pathway is also crucial for the normal functioning of the lungs and the digestive system; therefore, such attempts to shut down specific signaling pathways could be detrimental to the patient [73,76]. Similar inhibition of the CCL5/CCR5 pathway has been studied as a possible target for eliminating TAMs, and the FDA-approved HIV drug Maraviroc is currently being considered for cancer [77,78]. Among the other attempts to deplete TAM, targeting the colony-stimulating factor (CSF-1)/CSF-1R pathway, which is known to trigger TAM recruitment into tumors and polarization of these cells into the M2-like phenotype, has been considered. Some preclinical models showed that CSF-1R inhibition causes reduced TAMs and tumor growth [79]. However, other reports indicated that inhibition of the CSF-1/CSF-1R axis did not obliterate all macrophages but pushed the TAMs toward the M1-like phenotype triggering CD8^+^ T cell activation and inhibiting tumor progression [80,81]. Furthermore, CSF-1R blockade only caused a modest delay in tumor growth, thus yielding only limited therapeutic success [80,81]. An FDA-approved, small-molecule CSF-1R kinase inhibitor, BLZ945, did not eliminate macrophages in lung cancer but reprogrammed them into the M1 phenotype and triggered the recruitment of IFNγ-wielding NK and Tc cells and also IL-12-secreting dendritic cells with antitumor activity [82].

The macrophage repolarization strategy has also been tested by using agonists for the toll-like receptors TLR3, TLR4, and TLR7/8, which are known to repolarize M2-like TAMs into M1-like phenotypes to levels comparable to that achieved with lipopolysaccharides and IFNγ [83]. Another preclinical study was conducted using poly-ICLC (polyinosinic-polycytidylic acid), a TLR3 agonist, with promising results [84]. A relatively new strategy of repolarizing TAMs involves the cyclic guanosine monophosphate-adenosine monophosphate synthase (cGAS)-stimulated interferon gene (STING) pathway, which appears to be sensitive to cytosolic DNA, typically observed in tumor cells. This cGAS-STING signaling pathway launches innate immune responses, producing type I interferons, which trigger M1 polarization of TAMs and subsequent adaptive immune response [85,86,87]. A STING agonist, 5,6-dimethyl xanthenone-4-acetic acid (DMXAA) (a.k.a. Vadimezan), was used in clinical trials [88,89], however, the phase III clinical trial failed to show any positive outcome. Possible reasons were proposed for this failure, pointing to the species-specificity of the DMXAA, which may not activate human STING, and that DMXAA targets only highly vascularized cancers. In contrast, the ones included in the Phase III trial had normal vasculature. Furthermore, DMXAA may cause hypoxia after vasculature reduction, which would induce the production of the vascular endothelial growth factor (VEGF) and angiogenesis. Thus, DMXAA treatment and an angiogenesis inhibitor could prove more effective against cancers.

It should be noted that almost all of the studies involving the strategies of repolarizing the TAMs were combined with other treatments, such as checkpoint-inhibition immunotherapy, chemotherapy, or radiotherapy. Since the M1-polarized microglia and macrophages are known to cause recruitment and activation of NK cells and Tc cells, which have antitumor effects, most future studies will test “multi-therapy” rather than monotherapy. One more valuable message can be derived from the failed clinical trials: most therapeutics against diseases follow the general concept of targeting one signaling molecule since in vitro studies in test tubes and cultured cells are used to confirm the specificity of the targeting agent against that signaling molecule. In this process, a novel targeting molecule is synthesized and patented. However, after the FDA eventually approves this molecule to target a specific protein and treat a specific symptom, the same molecule is often found to also function on another target, allowing it to be repurposed for an unrelated condition. As a good example, the diabetes medication Metformin has been repurposed to treat Fragile X-syndrome-linked symptoms [90].

**Figure 2:**
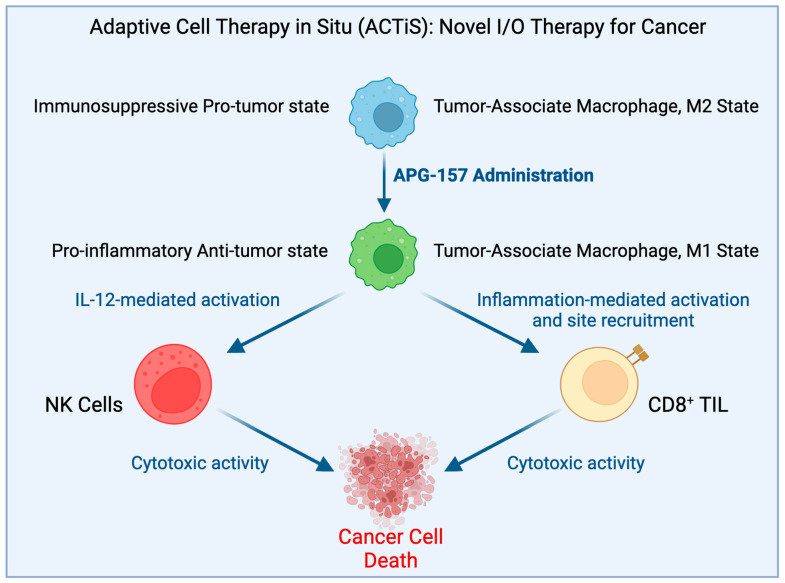
Proposed hypothesis for how APG-157 works.

Most beneficial compounds found in nature and in our diet are similarly pleiotropic, functioning on multiple targets (Figure 1). The major difference between the dietary compounds and the synthetic compounds is centered around the fact that none of the beneficial dietary compounds shut down one specific biochemical pathway completely. In sharp contrast, many synthetic compounds do so, which often causes injury to normal, noncancerous cells, thereby precipitating significant adverse effects. Although many such beneficial natural compounds are currently being studied in preclinical studies, they are rarely considered for clinical trials. In a double-blinded placebo-controlled Phase I clinical trial of 25 subjects, including an arm of biopsy-proven head and neck cancer patients, the subjects received a synergistic drug combination (APG-157) derived from the dietary spice turmeric [91]. The drug was delivered in a pastille form that enabled topical absorption into the tumors in the oral cavity and into the oropharyngeal tumor through salivary transport and systemic absorption through sublingual and buccal absorption. Thus, the drug was rapidly absorbed directly into the tumor and showed rapid systemic absorption [92]. This study used circulating plasma cell-free RNA (cf-RNA) as an effective indicator of drug response on tumor breakdown [90]. The promising observation made by this group included the upregulation of RNA transcripts bearing signatures of an inflammatory response, leukocyte activation, and upregulation of inflammatory cytokines in APG-157-treated patients but not in the healthy or placebo-treated patients. These changes indicate an immune response and a mobilization of immune cells triggered by the treatment. An especially striking observation was the increase in TNF-α response which points to an increase in tumor apoptosis. Since inflammatory cytokines secreted by immune cells in the TME play a vital role in TAMs’ repolarization into the M1 phenotype, the increase in TNF-α transcripts in cf-RNA observed in this case reflected M2 → M1 repolarization of TAMs in the TME (Figure 2). M1 macrophages release proinflammatory cytokines, such as TNF-α, along with IL-1β, and IL-6, to activate innate immunity and kill tumor cells [93]. The pleiotropic action of the drug was further confirmed by (i) the ability of the drug to reverse the cancer-driven dysbiosis of the oral microbiome, as measured by 16S RNA sequencing, and (ii) immunofluorescence of the tumor tissues before and after the drug administration showing immune system activation by recruitment of CD8+ Tc cells to the tumor as expected when TME experiences M2 to M1 reprogramming of TAM.

The cells and signaling activities of the innate immune system have been discussed in the preceding paragraphs. Still, it is equally important to understand that innate immunity also arises within aberrant cells, which are different from the innate immune cells, causing their self-elimination through apoptosis [94]. It is quite likely that such innate immunity within aberrant cells is one of the reasons why a defect in the nucleic acid sequence or structure rarely leads to cancer. Viewed from a different angle, continuous inflammatory signals released by such aberrant cells may also create a condition conducive to carcinogenesis [95].

Named as “R-loops”, cells acquire nucleic acid structures comprising an RNA–DNA hybrid and a non-template, single-stranded DNA. The R-loops have been implicated in human diseases, including repeat-expansion disorders, neurological syndromes, and cancer [96,97]. In cancer cells with mutations in, for example, the breast cancer predisposition gene *BRCA1*, which is known to code for a protein involved in DNA repair [94,98], a significant portion of the RNA–DNA hybrids exit the nucleus and accumulate in the cytoplasm. This gives rise to “innate immunity”, which can also occur in the presence of cytoplasmic DNA from pathogens. The signaling that results from such cytoplasmic DNA or RNA–DNA hybrids involves two major types of proteins, cGAS and the toll-like receptors TLR-3 and TLR-9, which selectively bind to cytoplasmic DNA hybrids and trigger downstream signals [99,100,101]. Although both cGAS and TLRs are expressed mainly by the innate immune cells, they are also expressed by the tumor cells. Using the classic cervical cancer cell line HeLa in culture, Crossley and coworkers achieved induction of cytoplasmic RNA–DNA accumulation by knocking down the RNA–DNA helicase (SETX) or the breast cancer gene *BRCA1*. Thus, they demonstrated that induction of cytoplasmic RNA–DNA hybrids sets off an innate immune response even in cancer cells, thereby triggering Ser386 phosphorylation of the interferon regulatory transcription factor 3 (IRF3), which in turn induces apoptosis [94]. The induction of cytoplasmic RNA–DNA hybrid levels also caused a dramatic increase in the signaling proteins interferon β (IFNβ), interferon-stimulated gene 15 (*ISG15*), *ISG20*, chemokine ligand 5 (CCL5), and tumor necrosis factor (TNF). In the presence of the cGAS inhibitor RU.521 or after depletion of TLR3, a sharp decrease in phosphorylated IRF3 and these downstream effectors was observed in the HeLa cells, thus establishing the involvement of cGAS and TLR3 in the RNA–DNA hybrid-triggered innate immune response. To further study the effect of the RNA–DNA hybrids in innate immune cells, Rigby and coworkers synthesized a 60-basepair RNA–DNA hybrid and transfected it into isolated and cultured dendritic cells [101]. Their experiments demonstrated that TLR9 selectively binds to the nucleic acid hybrid, thereby causing IRF3-mediated activation of type I interferons and boosting the secretion of cytokines such as IL-6 and IFN-α3. Finally, Boros-Oláh and coworkers considered the R-loop-forming genes as drug targets for cancer therapy [102]. In silico analysis by this group used The Cancer Genome Atlas (TCGA) to study 33 primary cancer types. To investigate the correlation between R-loop gene expression and survival rate among cancer patients, the authors used data from TCGA to generate Kaplan–Meier survival curves. In 70% of cases, low expression of R-loop genes, such as *RNASEH2A*, *THOC6*, *PRMT1*, and *P1F1*, was observed to be associated with prolonged survival of cancer patients with mesothelioma and a low expression of *FANCM* was linked to prolonged survival among breast cancer patients. However, in 30% of cases, high expression of R-loop genes, such as *TREX1* and *BUB3*, was associated with prolonged survival of patients with cervical squamous cell carcinoma and endocervical adenocarcinoma. For ten R-loop genes (*ATXN2*, *BRCA2*, *CARM1*, *DDX19A*, *RNASEH1*, *THOC2*, *THOC3*, *TOP1*, *U2AF1*, and *ZNF207*), long-term survival was observed only in the low-expressing group of patients. This study also reported an 80% association between the expression levels of R-loop genes in cancer cell lines and their sensitivity to chemotherapeutics approved by the US Food and Drug Administration (FDA). However, they also observed significant variability in drug interactions; for example, lung small cell carcinoma and ovarian cancer cells were sensitive to most of the drugs, however, B-cell leukemia, Hodgkin’s lymphoma, head, and neck cancer, and Ewing sarcoma cells were less susceptible to the FDA-approved chemotherapeutics.

## 7. Successes, Adverse Events, and Efforts to Avoid Them

The current “standard of care” involves mainly strategies of direct attack and killing of cancer cells in a tumor. In this strategy, the mutating cancer cells often develop chemoresistance, however, the chemotherapy-mediated killing of fast-dividing immune cells precipitates unwanted infections. Additionally, immunotherapy, currently used for many types of cancer, sometimes causes autoimmune attacks. A comprehensive analysis of immune checkpoint inhibitor therapy of 4489 patients with primary melanoma and a median age of 74.9 was recently reported. This study also had a follow-up survey, in which 1575 patients displayed immune-related adverse events (AE) [11]. Other AEs result from inhibiting an array of diverse signaling pathways, summarized elegantly in a few reviews [103,104]. As for successes, a report published by Merck for the PD-1 antibody drug Keytruda (pembrolizumab) in non-small cell lung carcinoma (NSCLC) showed an overall five-year survival (OS) rate of 23% in treatment-naïve patients (n = 101) and 15.5% OS in patients receiving prior treatment (n = 449). Among patients with PD-L1-expressing tumors, the OS was higher at 29% (n = 27) and 25% (n = 138), respectively [105].

As mentioned earlier, among the immune checkpoint inhibitors, several PD-1 antibodies, some CTLA-4 antibodies, and some PD-L1 antibodies have been used in clinical trials. Among these agents, atezolizumab, a PD-L1 inhibitor, appeared to have the best safety profile [106]. However, some patients treated with atezolizumab experienced chills, pyrexia, and flushing, possibly due to the activation of innate immunity by the intact human Fc region in this antibody. These relatively mild AEs were managed with paracetamol, antihistamine, and steroids only when required.

## 8. Summary and Concluding Remarks

In this review, we have attempted to give an overview of cancer therapy strategies at the preclinical and clinical levels, which mainly involve the innate and adaptive immune systems. First, we cite the work of Afshar-Sterle and coworkers showing that although deregulated expression of the *BCL6* oncogene is observed in many B-cell lymphoma patients, deliberate overexpression of this gene in mice does not cause lymphoma unless CD28- and Fas-ligand activities are simultaneously impaired in CD8+ Tc cells [5]. We also cited the work of Shi and coworkers; *knl1*-deletion-mediated DNA damage concomitantly triggers apoptosis and phagocytosis of neural progenitor cells by microglia [6]. Therefore, synchronous involvement of both innate and adaptive immune cells protects an organism from DNA mutation-evoked cancer. We have also discussed some promising strategies involving immunotherapy involving the empowerment of Tc cells and the difficulties experienced in the clinic with immunotherapy. Cognizant of the promise of using innate immune cells such as activated dendritic cells and NK cells in cancer therapy, several preclinical studies have been conducted, revealing that in the presence of pathogens, the dendritic cells secrete IFN1, which causes the activation of CD8+ Tc cells [61,62,63]. We have also discussed strategies to eliminate the tumor-promoting M2 macrophages and repolarizing them into the tumoricidal M1 phenotype. The first group of studies revealed that the inhibition of the (CSF-1)/CSF-1R pathway, which triggers TAM recruitment into tumor and M2-polarization of the recruits, only pushes the TAMs to the M1-like phenotype, which also causes CD8+ Tc cell activation [80,81,82]. A few relatively new methods of TAM repolarization were also discussed, using the cGAS-STING axis and the TLR3 agonist poly-ICLC [84,85,86,87,88,89]. In our preclinical studies of both GBM and peripheral cancers, we have noticed a profound role of TAMs in initiating a cascade of events involving activated NKp46+ NK cells and CD68+ Tc cells [15,19,20,23]. Thus, it can be concluded that the innate and adaptive immune systems work in close coordination. This notion must be front and center in designing safer and more effective cancer therapy strategies. We have argued that, in contrast to many synthetic CAs that completely shut off a specific signaling axis, thus causing adverse side effects, the most beneficial dietary anticancer compounds are pleiotropic and do not completely shut off any particular pathway. However, recently, they have rarely been considered for clinical studies. Two such studies, conducted recently by Basak and coworkers and Tosevska and coworkers, used a turmeric-based drug, APG-157, in head and neck cancer patients and measured cf-RNA to note leukocyte activation and the upregulation of transcripts bearing an inflammatory response and also a reversal of cancer-driven dysbiosis of the oral microbiome [91,92]. It is perhaps understood from a large number of attempts to develop an effective strategy for difficult-to-treat cancers that we may need to divert our attention from designing molecules to directly kill the cancer cells to empowering the immune system as a whole so that patients regain the ability to eliminate the mutated cells quickly before they cause cancer. For years, epidemiological studies have shown a link between cancer and diet. Still, we have continued to synthesize new antimetabolites and drugs to selectively activate some specific immune cells without making a concerted effort to take lessons from our diet and lifestyle and apply them to empower the human body to eliminate such aberrant cells. It is time that we change our approach to conquer many deadly cancers, such as pancreatic cancer and GBM.

## Data Availability

Data generated by the prior experiments have been published and cited in this review. Further details will be available upon request.
